# Comprehensive analysis of the influence of G-CSF on the biodistribution of ^18^F-FDG in lymphoma patients: insights for PET/CT scheduling

**DOI:** 10.1186/s13550-019-0546-1

**Published:** 2019-08-20

**Authors:** Magno Oliveira, Charline Lasnon, Catherine Nganoa, Anne-Claire Gac, Gandhi Damaj, Nicolas Aide

**Affiliations:** 1 Nuclear Medicine Department, CHU de Caen, Caen, France; 2 Nuclear Medicine Department, Centre François Baclesse, Caen, France; 3 INSERM 1199 ANTICIPE, Normandy University, Caen, France; 4 Haemalology Institute, CHU de Caen and Centre François Baclesse, Caen, France

**Keywords:** Lymphoma, PET/CT, ^18^F-FDG, G-CSF, Lenogastrim, Filgastrim, Pegfilgastrim, Sink effect, Deauville score

## Abstract

**Aims:**

(1) To perform a comprehensive analysis of the time elapsed between the last G-CSF injection and the PET/CT examination on the biodistribution of ^18^F-FDG, with emphasis on liver, spleen, and bone marrow uptake, and (2) to investigate whether an inversion of the liver to spleen ratio affects the Deauville scoring.

**Materials and methods:**

Retrospectively included were 74 consecutive diffuse large B cell lymphoma (DLBCL) patients referred for baseline and interim examinations and receiving immunochemotherapy with various G-CSF regimens. A comprehensive evaluation considering baseline metabolic active tumour volume (MATV), factors affecting liver uptake, the type of G-CSF, and the time elapsed between chemotherapy/G-CSF and interim PET/CTs was performed.

**Results:**

Mean (± SD) percentage variations between baseline and interim PET/CTs (i-PET/CT) for bone marrow (%Variation__BONE_), liver (%Variation__LIVER_) and spleen (%Variation__SPLEEN_) were equal to 32.0 ± 46.9%, 16.1 ± 42.8%, and 10.6 ± 51.1 %, respectively. %Variation__LIVER_ and %Variation__SPLEEN_ were higher in patients using lenograstim, but this was linked to lower uptakes at baseline and was therefore likely not due to G-CSF itself. The mean delay between G-CSF injection and i-PET/CT acquisition was not an independent explanatory variable for %Variation__BONE_, %Variation__LIVER_, and %Variation__SPLEEN_. On the contrary, %Variation__BONE_ and %Variation__SPLEEN_ were negatively correlated to the time-lapse between the end of chemotherapy and i-PET/CT: *ρ* = − 0.342 (*p* = 0.010) and *ρ* = − 0.529 (*p* < 0.0001), respectively. Patients with a time-lapse since the last injection of chemotherapy < 17 days displayed higher bone and spleen SUVmax_EARL_. %Variation__LIVER_ was positively correlated to baseline MATV: *ρ* = 0.243 (*p* = 0.039). Patients displaying a high baseline MATV ≥ 177 cc had significantly lower liver SUVmax_EARL_ at baseline. This difference was no longer observed at i-PET/CT, after tumours had shrunk.

**Conclusions:**

Neither the type of G-CSF used nor the time elapsed between its last injection and i-PET/CT examination independently influences bone, hepatic, or splenic uptakes at i-PET/CT. The major determinant for the occurrence of a bone or spleen hypermetabolism on i-PET/CT is the time elapsed between the chemotherapy and the examination, which should be maintained above 15 days. Inversion of the liver to spleen ratio appeared to be due to increased spleen hypermetabolism on i-PET/CT, making unlikely an impact on the Deauville scoring.

## Background

Amongst the factors thought to alter the ^18^F-FDG biodistribution in cancer patients receiving chemotherapy are granulocyte colony-stimulating factors (G-CSFs), which are used to reduce the incidence of neutropenia [1, 2]. The use of G-CSF close to the ^18^F-FDG PET/CT examination affects the ^18^F-FDG biodistribution, by significantly increasing the ^18^F-FDG uptake in the bone marrow and in the spleen [3, 4]. The proportion of Hodgkin’s lymphoma and non-Hodgkin’s lymphoma patients receiving G-CSF is high, as the chemotherapy regimens used tend to induce leucopenia. Several drugs are available (pegfilgrastim, filgrastim, and lenograstim), requiring 1 to 5 daily subcutaneous injections on average, starting 2 days after the end of chemotherapy.

G-CSFs act on the monocyte-macrophage haematopoietic cell lineage to stimulate progenitor proliferation, differentiation, and functional activation of the mature haematopoietic cells. Due to their renal clearance, filgrastim and lenogastrim are short-acting G-CSF and require daily injections. Pegfilgrastim has the same mechanism of action as filgrastim, but is pegylated and therefore has a reduced renal clearance, compared with that of filgrastim, prolonging the serum half-life of the drug in vivo and requiring a single injection.

The 2014 EANM guidelines for PET/CT tumour imaging [5, 6] and the ASCO recommendations for PET/CT imaging in lymphoma [7] recommend a time-lapse of 10 days between chemotherapy and PET/CT imaging. It is therefore a common practice to schedule interim PET/CT (i-PET/CT) and end-of-treatment PET/CT (EoT-PET/CT) not too close to the last G-CSF injection. This is however not always feasible in busy PET/CT centres and also because a chemotherapy regimen may be postponed for various reasons including insufficient blood count recovery. Consequently, some patients may be imaged shortly after their last chemotherapy and G-CSF injections, harbouring an increased bone marrow and spleen uptake. This results in an inversion of the liver to spleen ratio that could alter the post-treatment evaluation using the Deauville score (DS) [8]. Also affecting ^18^F-FDG biodistribution and potentially the DS is tracer sequestration in bone marrow, known as the sink effect. This phenomenon has been reported to reduce tracer availability in healthy tissues in patients with bulky neuroendocrine tumours [9]. In the case of lymphoma patients, this could affect the liver uptake and/or the residual tumour uptake.

The aims of the present study that focused on a homogeneous population of diffuse large B cell lymphoma patients scanned for interim evaluation after immune-chemotherapy were (1) to perform a comprehensive analysis of the time elapsed between the last G-CSF injection and the PET/CT examination on the biodistribution of ^18^F-FDG , with emphasis on liver, spleen, and bone marrow uptake, and (2) to investigate whether an inversion of the liver to spleen ratio affects the Deauville scoring.

## Materials and methods

### Patient recruitment

This study retrospectively included consecutive patients referred to our PET/CT unit for baseline and interim examinations (after 4 cycles of chemotherapy), diagnosed with DLBCL between December 2014 and December 2017. EoT-PET/CTs were not included, as these scans are usually performed several weeks after completion of treatment, when effects of G-CSF tend to be negligible. All selected patients had received R-CHOP (rituximab, cyclophosphamide, doxorubicin, vincristine, prednisone) or R-ACVBP (rituximab, doxorubicin, cyclophosphamide, vindesine, bleomycin, prednisone) and G-CSF. For each patient, age, gender, Ann Arbor staging, baseline bone, spleen or liver involvement, Deauville scoring at interim PET/CT, the number of injections of G-CSF and date of the last one, the type of G-CSF, and the date of the last injection of chemotherapy were recorded from the patients’ medical files. Criteria of exclusion were chemotherapy other than R-CHOP or R-ACVBP, history of splenectomy, interim examinations not performed after 4 cycles, and no use of G-CSF.

Approval to collect data for our study was validated by the national committee for data privacy, the National Commission on Information Technology and Liberty (CNIL), with registration no. 2204611 v 0, and patients’ consent to use anonymised data for research purposes was sought on an opt-out basis.

### PET/CT acquisition and reconstruction parameters

PET/CT examinations were performed as per the EANM guidelines for PET/CT tumour imaging [5, 6].

Quantitative data presented in this study are EARL-compliant. Patients fasted at least 6 h before intravenous injection of ^18^F-FDG and were scanned on a Biograph TrueV system (Siemens Heathineers) from the skull base to the mid-thighs. A free-breathing CT acquisition (60 mAs, 130 kVp, pitch 1, and 6 × 2 mm collimation) was followed by a PET/CT acquisition with time per bed position of 160 and 220 s for normal weight (BMI ≤ 25 kg/m^2^) and overweight patients (BMI > 25 kg/m^2^), respectively.

PET/CT studies were planned to be performed 60 ± 5 min post-injection. Extraction of data from the PET/CT DICOM headers showed the following information: mean injected activity ± SD was 4 ± 0.1 MBq/kg. Mean delay between injection and acquisition ± SD was 59.3 ± 5.2 min on baseline and 4 ± 0.2 MBq/kg and 60.3 ± 5.0 min on interim TEP, respectively.

Raw data were reconstructed with point spread function (PSF) modeling (HD; TrueX, Siemens Heathineers), with 3 iterations and 21 subsets and no filtering. Matrix size was 168 × 168 resulting in voxels of 4.07 × 4.07 × 4 mm.

### PET/CT analysis

All PET/CT examinations were reviewed on Syngo.via Software (Siemens Medical Solution). A 6.3-mm Gaussian filter, determined as per the EARL accreditation programme, was applied using the EQ.PET software [10, 11]. For each PET/CT examination, maximum standardized uptake values (SUVmax) were measured as follows:

- For liver and spleen, an automatic 3-cm-diameter volume of interest (VOI) was placed in the right liver lobe, avoiding liver or spleen lesions in the case of tumour involvement. A manually set VOI fitting the size of the spleen was used in cases where spleen was smaller than the 3 cm VOI.
- For bone marrow assessment, a VOI of 2 cm was placed on the sacral promontory, avoiding sacral foramens.

Liver mean Hounsfield units (HU) were measured to seek hepatic steatosis [12]. Baseline metabolic active tumour volume (MATV) was measured for each patient using a 41% isocontour method as per the EANM guidelines. In the case of multiple lesions, MATVs of all lesions were summed.

### Statistical analysis

Quantitative data are presented as mean ± standard deviation (SD). Baseline and interim PET/CT technical PET/CT characteristics (injected activity, glycaemia, delay), as well as patient’s BMI and liver densities, were compared using the non- parametric Wilcoxon test for paired samples. To seek a difference between %Variation__BONE_, %Variation__LIVER_, or %Variation__SPLEEN_ amongst the three types of G-CSF, the Kruskal-Wallis test was used. Spearman’s correlations were performed to seek a link between %Variation__BONE_, %Variation__LIVER_, or %Variation__SPLEEN_ and BMI percentage variation (%Variation__BMI_), glycaemia percentage variation (%Variation__Glycaemia_), and baseline MATV as well as the time elapsed between the end of chemotherapy or the last G-CSF injection and interim PET/CT acquisition. Spearman’s correlation was also used to search for a correlation linking the time-lapse between chemotherapy or G-CSF last injection and interim PET/CT. To seek a difference between SUVmax_EARL_ amongst different groups of patients, the non-parametric Mann-Whitney or Kruskal-Wallis tests were used as appropriate. When needed, complementary multivariate analysis was performed using ANCOVA. With regard to the inversion of the liver to spleen ratio, differences in the time-lapse between the end of chemotherapy or the last G-CSF injection and interim PET/CT in patients with this pattern versus those without were tested using the Mann-Whitney test. Moreover, difference in the repartition of G-CSF type used between these two groups of patients was tested using Fischer’s exact test.

## Results

### Population and PET/CT characteristics

Population characteristics can be seen in Table 1, and PET/CT characteristics in Table 2. On paired comparison, the injected activities for baseline and interim PET/CTs were not significantly different; neither was the time-lapse between injection and acquisition and liver densities. Mean BMI and glycaemia were significantly different between baseline and interim PET/CT acquisition (Table 2, *p* < 0.0001 and *p* = 0.044, respectively). The mean bone uptake %variation between baseline and interim PET/CTs (%Variation__BONE_) was equal to 32.0 ± 46.9% (*n* = 57), the mean liver uptake %variation (%Variation__LIVER_) was equal to 16.1 ± 42.8% (*n* = 73), and the spleen uptake %variation (%Variation__SPLEEN_) was equal to 10.6 ± 51.1% (*n* = 66). None of them were influenced by the %Variation__BMI_: *ρ* = 0.210 (*p* = 0.117) for %Variation__BONE_, *ρ* = − 0.071 (*p* = 0.548) for %Variation__LIVER_, and *ρ* = 0.151 (*p* = 0.227) for %Variation__SPLEEN_. None of them were influenced by the %Variation__Glycaemia_: *ρ* = − 0.039 (*p* = 0.772) for %Variation__BONE_, *ρ* = 0.138 (*p* = 0.244) for %Variation__LIVER_, and *ρ* = 0.012 (*p* = 0.921) for %Variation__SPLEEN_. The mean time-lapse between the end of chemotherapy and i-PET/CT for the whole series of patients (*n* = 74) was equal to 16 ± 5 days (median = 17 days), and the mean time-lapse between G-CSF injection and i-PET/CT was equal to 8 ± 5 days (median = 8 days). There was a significant correlation between these two variables (*ρ* = 0.629, *p* < 0.0001).

**Table 1:** Patients’ characteristics (*n* = 74)

Characteristics	
Age (year), mean **±** SD **[**min-max**]**	60 ± 14 [21;80]
Sex, *n* (%)	
Female	35 (47.3)%
Male	39 (52.7)%
Age-adjusted IPI, *n* (%)	
0	8 (10.8)%
1	28 (37.8)%
2	24 (32.4)%
3	14 (19.0)%
Deauville score at i-PET/CT, *n* (%)	
1	23 (31.1)%
2	13 (17.6)%
3	11 (14.9)%
4	13 (17.6)%
5	14 (18.9)%
Growth factor type (G-CSF), *n* (%)	
Filgrastim	52 (70.3)%
Lenograstim	10 (13.5)%
Pegfilgrastim	12 (16.2)%
First line of chemotherapy, *n* (%)	
R-CHOP	68 (91.9)%
R-ACVBP	6 (8.1)%
Bone involvement*, *n* (%)	17 (23.0)%
Spleen involvement*, *n* (%)	8 (10.8)%
Liver involvement*, *n* (%)	1 (1.3)%
Baseline MATV (cc), mean **±** SD	449.4 (732.0)%

*IPI* International Prognostic Index, *i-PET/CT* interim PET/CT, *MATV* metabolic active tumour volume

*Based on PET/CT analysis

**Table 2:** PET/CT characteristics

Characteristics (mean ± SD)	Baseline PET/CT	Interim PET/CT	*p* value
Injected dose (MBq/kg)	4.0 ± 0.2	3.9 ± 0.4	0.132
Post-injection time (min)	59.2 ± 5.1	59.7 ± 4.9	0.602
BMI (kg/m^2^)	26.6 ± 5.6	25.7 ± 5.5	*< 0.0001*
Glycaemia (g/L)	1.00 ± 0.15	1.05 ± 0.19	*0.044*
Liver density (HU)	49.4 ± 10.1	48.8 ± 12.5	0.721

### Bone %variations between baseline and interim PET/CTs

This analysis was performed on the 57 patients with no bone involvement at baseline and interim PET/CT examinations.

**Fig. 1:**
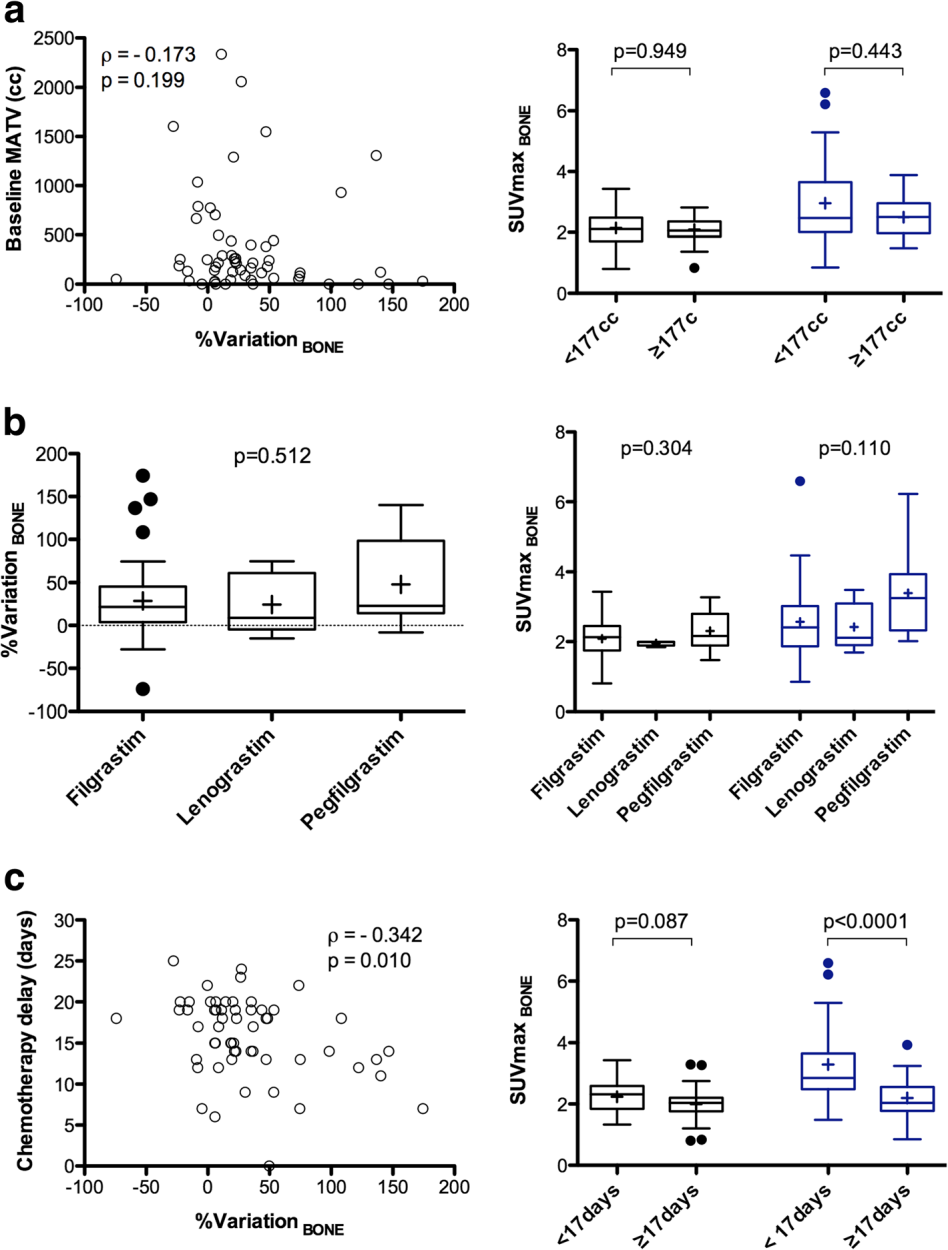
Percentage variation (right panels) or absolute value (left panels) for bone marrow uptake between baseline and interim PET/CT, depending on the metabolic active tumour volume (MATV) on baseline scan (**a**), the type of G-CSF (**b**), and the time-lapse between the last injection of chemotherapy and the PET/CT examination (**c**)

%Variation__BONE_ between baseline and interim was not correlated to the baseline MATV (*p* = 0.199, Fig. 1a). Moreover, there was no statistically significant difference in bone %variations between progressive disease patients (*n* = 5) and non-progressive patients: 45.2 ± 74.8% versus 30.8 ± 44.3%, *p* = 0.924. %Variation__BONE_ was not significantly different depending on which type of G-CSF was used (*p* = 0.512, Fig. 1b). %Variation__BONE_ was negatively correlated to the time elapsed between the end of chemotherapy and i-PET/CT acquisition but not correlated to the mean delay between G-CSF injection and interim PET/CT acquisition: *ρ* = − 0.342 (*p* = 0.010) and *ρ* = − 0.208 (*p* = 0.12) (Fig. 1c). At interim PET/CT, patients with a time-lapse between the end of chemotherapy and i-PET/CT acquisition < 17 days (representing the median value of the data) displayed higher bone SUVmax_EARL_ than the other patients (*p* < 0.0001). Of note, there were no significant differences in bone SUVmax_EARL_ at baseline between these two groups of patients (*p* = 0.087).

**Fig. 2:**
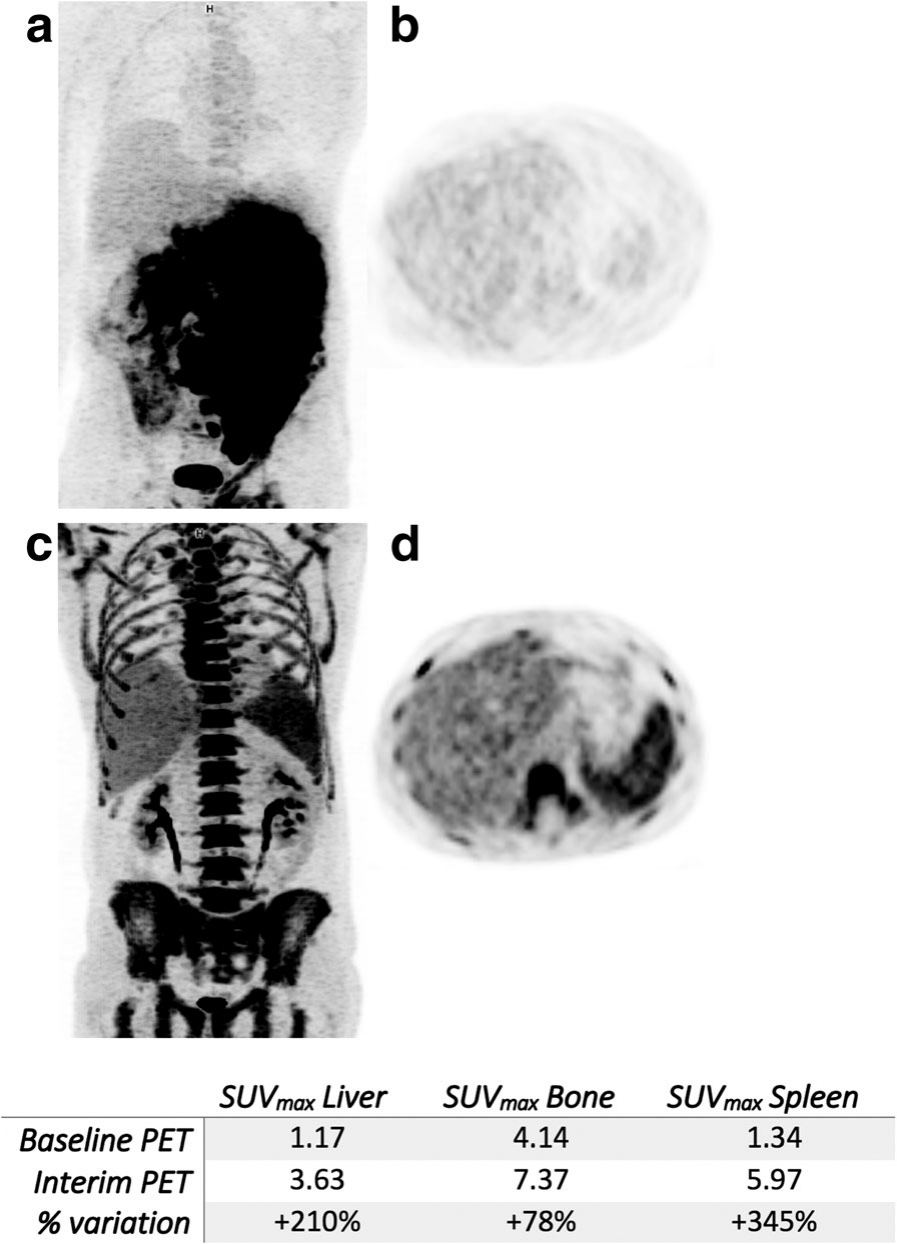
Fifty-two-year-old male patients imaged at baseline (**a**, **b**) and after 4 cycles of R-ACVBP (**c**, **d**). Interim PET/CT was performed 7 days and 6 days following the last injection of chemotherapy and G-CSF, respectively. The injected G-CSF was lenograstim, at a dosage of 34MUI daily for a 4-day period. In this case, low hepatic ^18^F-FDG uptake can be observed at baseline because of a tracer sequestration in the bulky mass. This pattern resolves after the tumour had shrunk on interim PET/CT, but because of a short time-lapse between the last chemotherapy injection and interim PET/CT, an increased bone marrow and splenic uptakes are observed

**Fig. 3:**
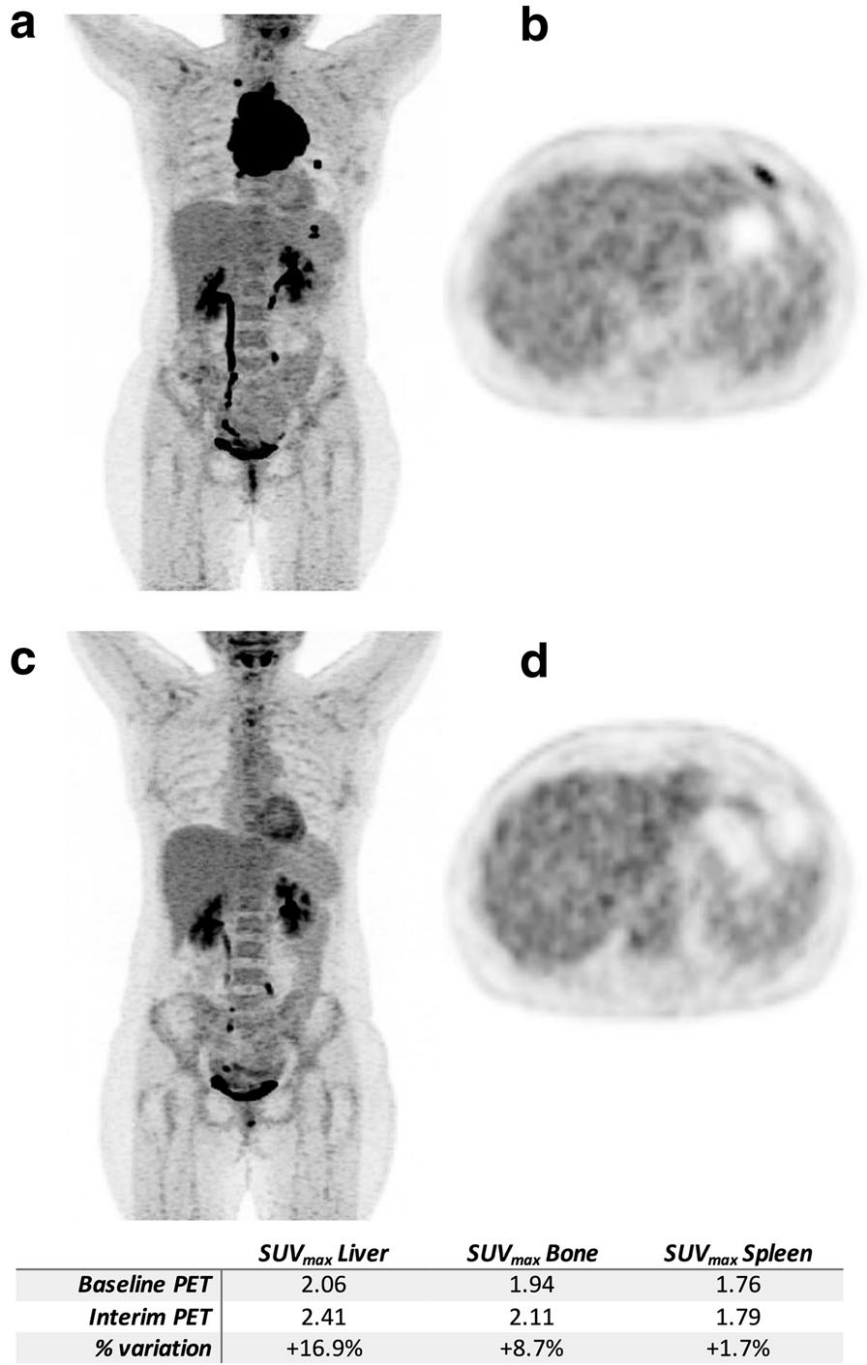
A 47-year-old female patient imaged at baseline (**a**, **b**) and after 4 cycles of R-ACVP (**c**, **d**). Interim PET/CT was performed 17 days and 12 days following the last injection of chemotherapy and G-CSF, respectively. The injected G-CSF was lenogastrim, at a dosage of 34MUI daily for a 5-day period. Thanks to a time-lapse between the last chemotherapy injection and interim PET/CT, no significant increased bone marrow or splenic uptake are observed

Representative images of patients with a short (< 17 days) or long time-lapse (≥ 17 days) between the end of chemotherapy and i-PET/CT acquisition scan can be seen on Figs. 2 and 3, respectively.

### Liver %variations between baseline and interim PET/CTs

This analysis was performed on the 73 patients with no liver involvement at baseline and interim PET/CT examinations.

**Fig. 4:**
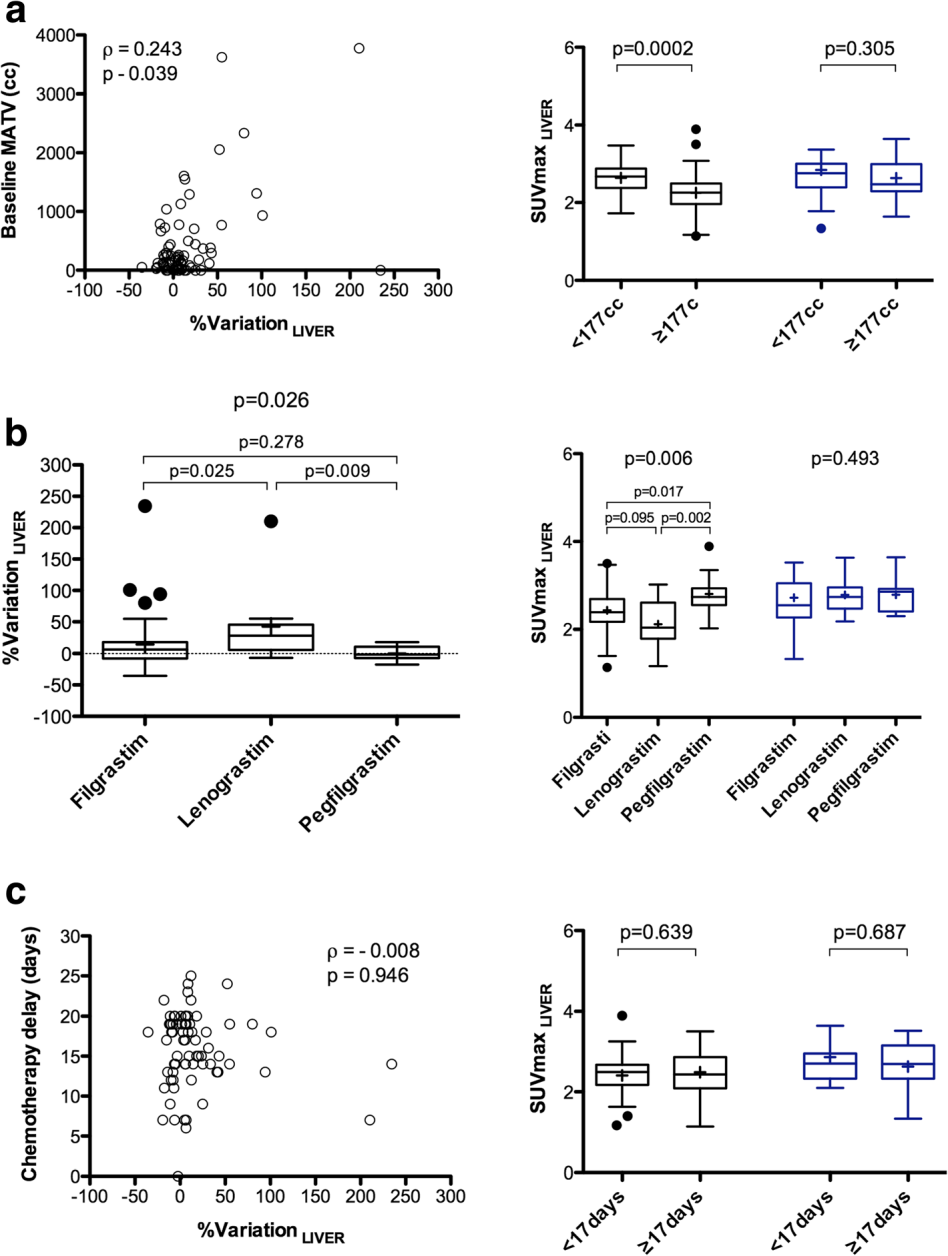
Percentage variation (right panels) or absolute value (left panels) for liver uptake between baseline and interim PET/CT, depending on the metabolic active tumour volume (MATV) on baseline scan (**a**), the type of G-CSF (**b**), and the time-lapse between the last injection of chemotherapy and the PET/CT examination (**c**)

%Variation__LIVER_ between baseline and interim was positively correlated to the baseline MATV (*ρ* = 0.243, *p* = 0.039, Fig. 4a). Moreover, %Variation__LIVER_ was lower in progressive disease patients (*n* = 5): − 8.7 ± 11.1% versus 17.9 ± 43.7%, *p* = 0.013. Patients displaying a high baseline MATV ≥ 177 cc (representing the median of the data) had significantly lower liver SUVmax_EARL_ at baseline (*p* = 0.0002). This difference was no longer observed at interim PET/CT (*p* = 0.305). %Variation__LIVER_ was significantly different depending on which type of G-CSF was used (*p* = 0.026). %Variation__LIVER_ was higher when using lenograstim as compared to filgrastim or pegfilgrastim, with a mean %variation of 42.6 ± 62.0%, 14.6 ± 41.4%, and 0.2 ± 10.8%, respectively. Baseline liver SUVmax_EARL_ were significantly lower for patients who received lenograstim, whereas there was no significant difference at interim PET/CT (Fig. 4b). %Variation__LIVER_ was not correlated to the time elapsed between the end of chemotherapy and i-PET/CT acquisition (*p* = 0.946, Fig. 4c) nor to the mean delay between G-CSF injection and interim PET/CT acquisition (*p* = 0.870).

On multivariate analysis accounting for baseline MATV and the type of GCSF used, baseline MATV was the only independent explanatory variable for %Variation__LIVER_ (*p* < 0.0001).

### Spleen %variations between baseline and interim PET/CTs

**Fig. 5:**
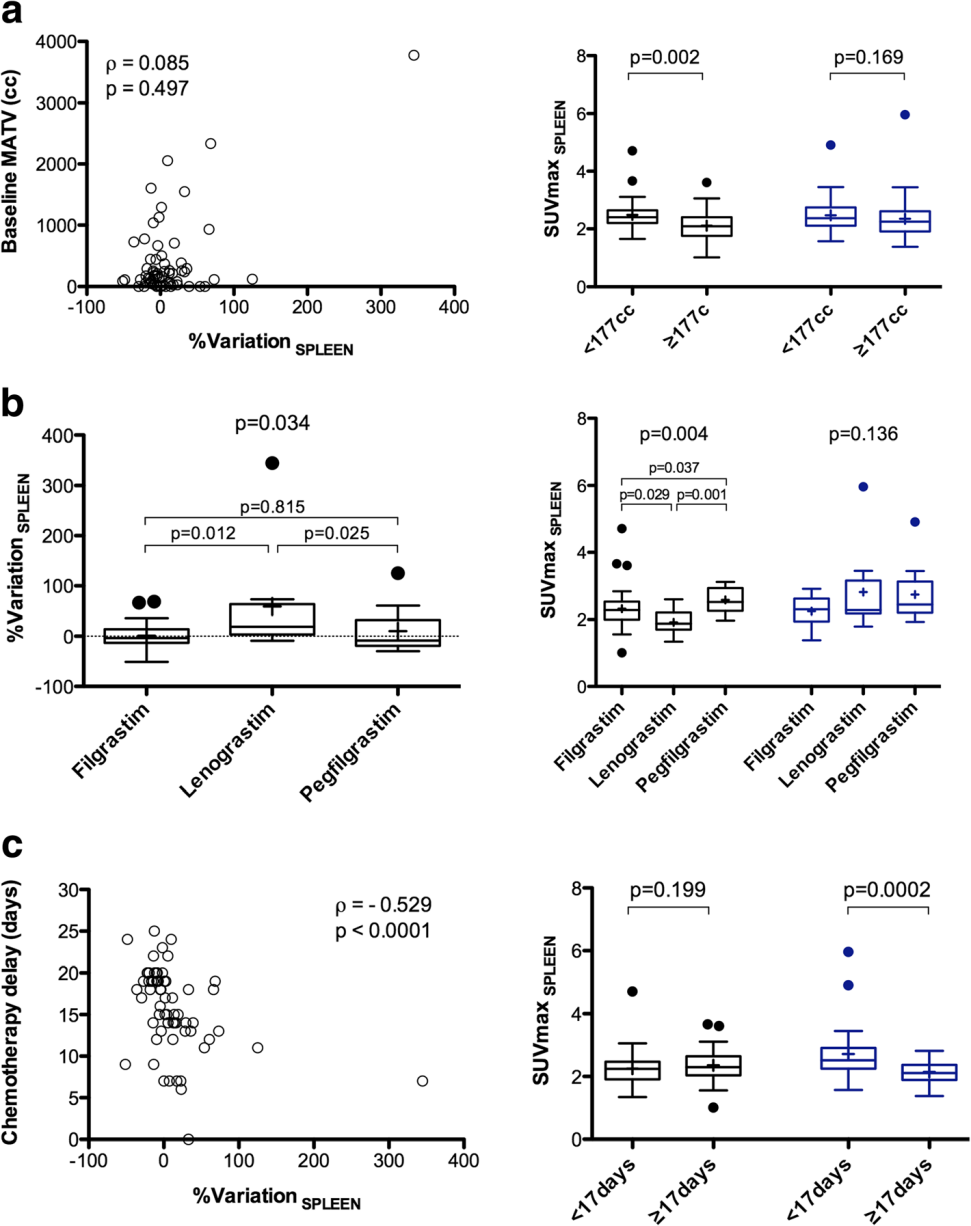
Percentage variation (right panels) or absolute value (left panels) for spleen uptake between baseline and interim PET/CT, depending on the metabolic active tumour volume (MATV) on baseline scan (**a**), the type of G-CSF (**b**) and the time-lapse between the last injection of chemotherapy and the PET/CT examination (**c**)

This analysis was performed on the 66 patients with no spleen involvement at baseline and interim PET/CT examinations. %Variation__SPLEEN_ between baseline and interim was not correlated to the baseline MATV (*ρ* = 0.085, *p* = 0.497, Fig. 5a). There was no statistically significant difference in %Variation__SPLEEN_ between progressive disease patients (*n* = 5) and non-progressive patients: − 5.6 ± 17.6% versus 11.9 ± 52.8%, *p* = 0.45. %Variation__SPLEEN_ was significantly different depending on which type of G-CSF was used (*p* = 0.034). %Variation__SPLEEN_ was higher when using lenograstim as compared to filgrastim or pegfilgrastim, with a mean %variation of 58.9 ± 110.3%, 1.0 ± 24.1%, and 10.3 ± 44.8%, respectively (Fig. 5b). Baseline spleen SUVmax_EARL_ were significantly lower for patients who received Lenograstim, whereas there was no significant difference at interim PET/CT. %Variation__SPLEEN_ was negatively correlated to the time elapsed between the end of chemotherapy and i-PET/CT acquisition (*ρ* = − 0.529, *p* < 0.0001, Fig. 5c) as well as to the mean time-lapse between G-CSF injection and interim PET/CT acquisition (*ρ* = − 0.343, *p* = 0.005). At interim PET/CT, patients with a time-lapse between the end of chemotherapy and i-PET/CT acquisition < 17 days (representing the median value of the data) displayed higher spleen SUVmax_EARL_ than the other patients (*p* = 0.0002). Of note, there were no significant differences in spleen SUVmax_EARL_ at baseline between these two groups of patients (*p* = 0.199). Representative images of patients with a short (< 17 days) or long time-lapse (≥ 17 days) between the end of chemotherapy and i-PET/CT acquisition scan can be seen on Figs. 2 and 3, respectively.

On multivariate analysis accounting the type of GCSF used**,** the time elapsed between the end of chemotherapy and i-PET/CT acquisition, and the mean time-lapse between G-CSF injection and interim PET/CT acquisition, the use of lenograstim and the time-lapse between the end of chemotherapy and i-PET/CT acquisition were independent explanatory variables for %Variation__SPLEEN_ with *p* value 0.007 and 0.033, respectively.

### Occurrence of inversion of the hepatosplenic ratio

This analysis was performed on the 65 patients with no spleen or liver involvement at baseline and interim PET/CT examinations.

Inversion of the hepatosplenic ratio occurred in 17 patients (26.1%). Patients with an inversion of the liver-to-spleen ratio had lower time-lapse between the end of chemotherapy and i-PET/CT acquisition as compared to others: 11 ± 5 days versus 18 ± 4 days, respectively (*p* < 0.0001). There was no significant difference in the type of G-CSF used between patients displaying an inversion of the liver-to-spleen ratio and those who did not (*p* = 0.218).

## Discussion

G-CSF are recommended after the two main chemotherapy regimens used to treat DLBCL, namely R-CHOP and R-ACVB, and have been reported to induce increased uptake in the spleen and in the bone marrow [3], the latter also being affected by normal regeneration following chemotherapy. Previous studies reported effects of G-CSF on the biodistribution of ^18^F-FDG at a time when only one type of G-CSF was available (pegfilgrastrim [3]), and did not account for the time elapsed between both the chemotherapy and the last injection of G-CSF and that PET/CT scan. Indeed, while the physiopathological basis of G-CSF makes obvious the link between this treatment and the observed increased uptake in the spleen [13], increased bone marrow uptake could be due to either G-CSF and/or bone marrow regeneration. Sequestration of ^18^F-FDG within the skeleton could alter residual tumour uptake and therefore the Deauville scoring. Given the observed variability in the type and duration of G-CSF treatment, a better knowledge of the phenomenon responsible for an altered bone marrow and spleen biodistribution and the duration of these patterns would be useful for a better scheduling of patients’ scans in the busy PET/CT unit. It would also help in deciding whether a PET/CT scan has to be postponed when a patient shows up early after the last injection of G-CSF.

In our study, lenograstim was associated with a higher percentage of variation in liver and spleen between baseline and interim PET/CT. However, the variation in the liver and spleen was linked to a lower uptake in the two organs at baseline, likely due to tumour sequestration in bulky tumour masses, and was therefore not due to the G-CSF itself. This hypothesis is supported by the fact that we found that %Variation__LIVER_ was lower in progressing patients compared to the non-progressing patients.

The time elapsed between the last injection of G-CSF and the interim PET/CT examination appeared less decisive than the time-lapse between the end of chemotherapy and the i-PET/CT examination, even if a strong correlation was observed. Indeed, no association between the variation in bone marrow, liver, and spleen and the time-lapse between the last injection of G-CSF and the interim PET/CT examination was found on multivariate analysis. On the other hand, a delay inferior to 17 days between the end of chemotherapy and the i-PET/CT examination led to increased bone and spleen uptakes on interim PET/CT images. These findings have practical consequences when planning PET/CT examinations; as an a priori knowledge of the type and dose of G-CSF, as well as patients’ compliance to injections is difficult to apprehend, scheduling PET/CT scans at least 15 days after the last injection of chemotherapy may be recommended. Of note, guidelines for PET/CT imaging in lymphoma patients recommend a 10-day delay after chemotherapy [7], which appears insufficient based on our data. Indeed, our results show that a time-lapse of 15 days could allow to eliminate the majority (9/12, 75%, Fig. 1c) of patients displaying a percentage of variation in the bone uptake greater than 50%, while taking the 10-day time-lapse would identify only a few of these patients (3/12, 25%, Fig. 1c).

Of note, despite its longer half-life compared to filgastrim and lenogastrim due to its PEGylated nature, we did not observe larger variations in bone marrow and spleen uptake in the group of patients having received pegfilgastrim. In the study from Jacene et al. [3] involving breast cancer patients receiving docetaxel plus pegfilgastrim, the time-lapse between the last injection of chemotherapy and PET/CT scan was 7 ± 0.7 days, and the time-lapse between the last injection of GCSF and PET/CT scan was 6 ± 0.7 days, compared to 16 ± 5 and 8 ± 5 days in our study, respectively. We feel likely that the greater increase in bone marrow and spleen they observed^1^ compared to our data (%Variation__BONE_ and %Variation__SPLEEN_ equal to 244.6 ± 127.3% and 171.3 ± 60.6% versus 32.0 ± 46.9% and 10.6 ± 51.1, respectively) is related to a shorter time-lapse post-chemotherapy.

On multivariable analysis, percentage variation in the liver was only influenced by the baseline MATV via the well-known sink effect that led to low liver uptake at baseline, this phenomenon being reversible after chemotherapy, when bulky tumour masses had shrunk, as shown in Fig. 4. Interestingly, hepatosplenic ratio inversion, which is only associated with a shorter time-lapse between the end of chemotherapy and the i-PET/CT acquisition, is therefore only due to an increase in spleen uptake. Lower ^18^F-FDG hepatic uptake in lymphoma patients due to a tumour sink effect has already been suspected by Wu et al. [14]. Another confounding factor potentially decreasing liver metabolism is liver steatosis [12]. However, in the present series, steatotic liver was only observed in 14 patients (18.9%) and liver densities were not different between baseline and interim PET/CT examinations, meaning that %Variation__LIVER_ is certainly not biased by steatosis. Therefore, overall, inversion of the hepatosplenic ratio is unlikely to have any influence on the Deauville score determination. However, the Deauville scoring could still be influenced by a sink effect [9] due to tracer sequestration in the bone marrow; Teoh et al. predicted the tumour uptake to be reduced by a maximum of 11.5 % in the case of G-CSF-induced hypermetabolism in the bone marrow [15]. Based on all these findings together, maintaining the time-lapse between the chemotherapy and the PET/CT examination greater than 15 days appears to be a simple way of avoiding disturbance in ^18^F-FDG biodistribution and detrimental effect on the Deauville scoring in interim PET/CT.

## Conclusion

Neither the type of G-CSF used nor the time elapsed between its last injection and interim PET/CT examination independently influences bone, hepatic, or splenic uptakes. The liver uptake is not influenced by any of these factors, and therefore, the occurrence of a hepatosplenic ratio inversion is only due to spleen hypermetabolism, making unlikely an impact on the Deauville scoring. The major determinant for the occurrence of a bone or spleen hypermetabolism on interim PET/CT is the time-lapse between the chemotherapy and the PET/CT examination, which should be maintained greater than 15 days.

## Data Availability

The datasets used and/or analysed during the current study are available from the corresponding author upon request.

## Abbreviations

%Variation__BMI_: Percentage variation between baseline and interim PET/CTs for BMI
%Variation__BONE_: Percentage variation between baseline and interim PET/CTs for bone marrow
%Variation__Glycaemia_: Percentage variation between baseline and interim PET/CTs for glycaemia
%Variation__LIVER_: Percentage variation between baseline and interim PET/CTs for liver
%Variation__SPLEEN_: Percentage variation between baseline and interim PET/CTs for spleen
^18^F-FDG: 18F-Fluorodeoxyglucose
BMI: Body Mass Index
DLBCL: Diffuse large B cell lymphoma
DS: Deauville scoring
Eot-PET/CT: End-of-treatment PET/CT
G-CSF: Granulocyte colony-stimulating factor
HU: Hounsfield unit
i-PET/CT: Interim PET/CT
MATV: Metabolic active tumour volume
PET/CT: Positron emission tomography/computed tomography
VOI: Volume of interest

## Footnotes

1 Data computed from Table 2 of reference [3].
